# Supplementary material for: Conditional chemoconnectomics (cCCTomics) as a strategy for efficient and conditional targeting of chemical transmission
Source: eLife. 2024 Apr 30;12:RP91927. doi: 10.7554/eLife.91927 (PMC11060718; doi:10.7554/eLife.91927)
Supplement: Figure 3—source data 3. [file elife-91927-fig3-data3.zip › Figure 3 source data3 part 1.zip!/Figure 3-Source data 3-part1/1071-1-CRISPResso_Report_osfbjvga.zip!/CRISPResso_on_1071_2/9.Alleles_frequency_table_around_sgRNA_gaccgtctcaccgtcaagat.pdf]

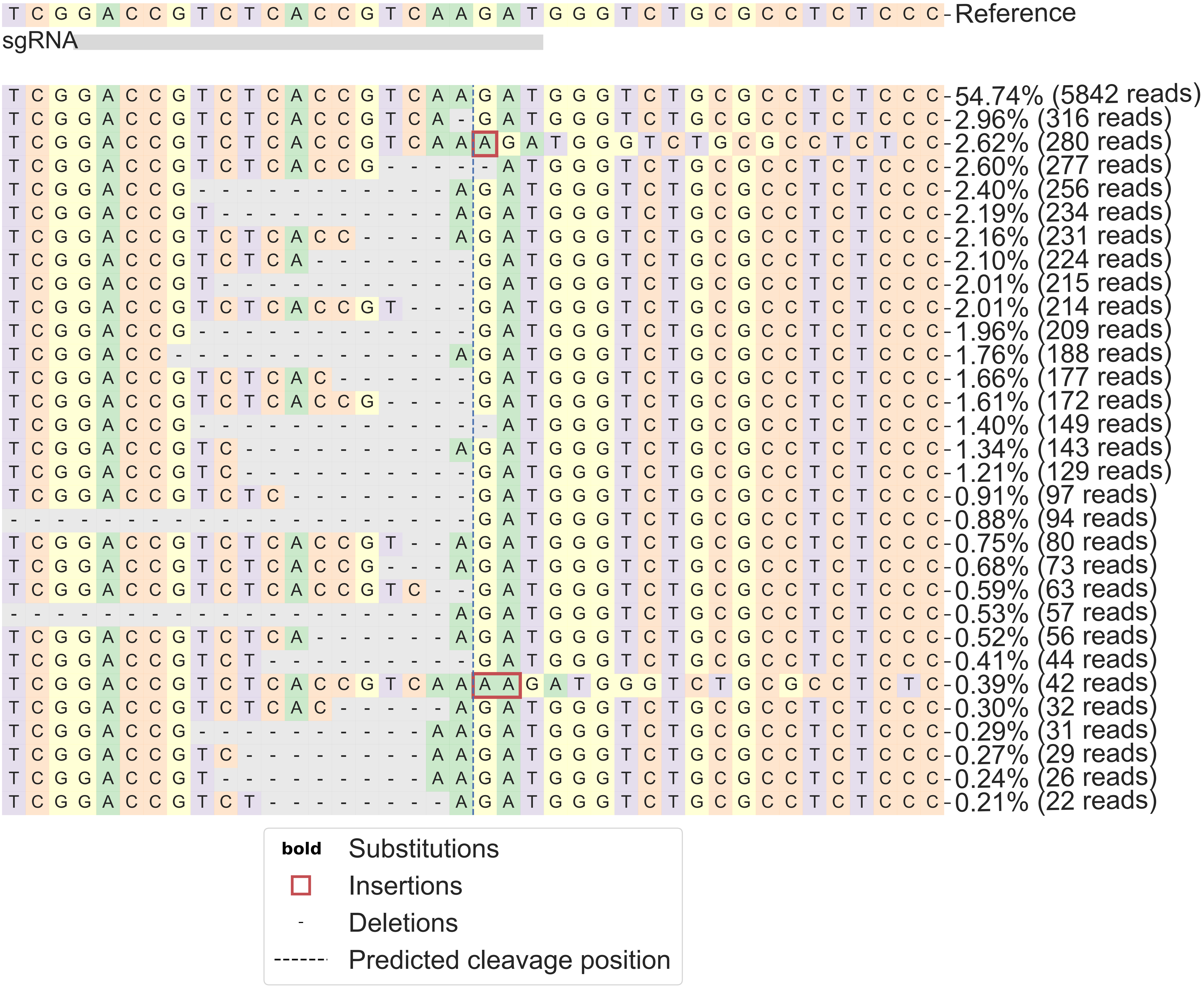
TCGGACCGTCTCACCGTCAAGATGGGTCTGCGCCTCTCCC Reference
sgRNA
54.74% (5842 reads)
2.96% (316 reads)
2.62% (280 reads)
2.60% (277 reads)
2.40% (256 reads)
2.19% (234 reads)
2.16% (231 reads)
2.10% (224 reads)
2.01% (215 reads)
2.01% (214 reads)
1.96% (209 reads)
1.76% (188 reads)
1.66% (177 reads)
1.61% (172 reads)
1.40% (149 reads)
1.34% (143 reads)
1.21% (129 reads)
0.91% (97 reads)
0.88% (94 reads)
0.75% (80 reads)
0.68% (73 reads)
0.59% (63 reads)
0.53% (57 reads)
0.52% (56 reads)
0.41% (44 reads)
0.39% (42 reads)
0.30% (32 reads)
0.29% (31 reads)
0.27% (29 reads)
0.24% (26 reads)
0.21% (22 reads)
bold Substitutions
Insertions
Deletions
Predicted cleavage position
